# Preliminary screening of urinary host protein biomarkers for Schistosomiasis haematobium: A proteome profiling study identifying candidate diagnostic targets in school-aged children

**DOI:** 10.1371/journal.pntd.0013429

**Published:** 2025-08-25

**Authors:** Yiyun Liu, Saleh Juma, Qingkai Xue, Mingzhen He, Said Mohammed Ali, Khamis Seif Khamis, Mchanga Mohd Suleiman, Mayda Salim Hamad, Mgeni Abdalla Khamis, Hongxia Zhao, Xin Dong, Kun Yang, Yuzheng Huang

**Affiliations:** 1 National Health Commission Key Laboratory of Parasitic Disease Control and Prevention, Jiangsu Provincial Key Laboratory on Parasite and Vector Control Technology, Jiangsu Institute of Parasitic Diseases, Wuxi, Jiangsu, China; 2 School of Public Health, Nanjing Medical University, Nanjing, Jiangsu, China; 3 Suining Center for Disease Control and Prevention, Suining, Sichuan, China; 4 Neglected Tropical Diseases Program, Ministry of Health, Chake-Chake, Pemba, United Republic of Tanzania,; 5 Changzhou Center for Disease Control and Prevention, Changzhou, Jiangsu, China; 6 Public Health Laboratory-Ivo de Carneri, Chake-Chake, Pemba, United Republic of Tanzania,; 7 School of Medicine, Shanghai University, Shanghai, China; 8 Zhanjiang institute of clinical medicine, Central People’s Hospital of Zhanjiang, Zhanjiang, Guangdong, China; Washington University in St Louis School of Medicine, UNITED STATES OF AMERICA

## Abstract

Schistosomiasis is a major public health challenge and a globally neglected tropical disease. *Schistosoma haematobium*, the causative agent of urogenital schistosomiasis, is endemic in African countries; with school-aged children ages 7–15 years being the most vulnerable population. Current diagnostic methods rely on microscopy to identify parasite eggs in urine; which is labor-intensive, requires specialized skills, and often lacks sensitivity, especially in mild infections. To address these limitations, we explored host disease-related biomarkers as a promising avenue for advancing diagnosis and detection. We recruited 135 children ages 7–15 years from Zanzibar, a known transmission hotspot, and used data-independent acquisition (DIA) proteomics combined with machine learning to identify potential host protein biomarkers in urine samples from individuals infected with *Schistosoma haematobium*. Proteomic analysis identified 823 common host proteins in urine samples from the infected group. Machine learning algorithms highlighted candidate discriminative proteins; which were validated using enzyme-linked immunosorbent assays (ELISA). Machine learning emphasized SYNPO2, CD276, α2M, LCAT, and hnRNPM as the most discriminating biomarkers for *Schistosoma haematobium* infection. ELISA validation confirmed the differential expression trends of these proteins, while machine learning further validated LCAT and α2M, underscoring their diagnostic potential. Our study focused on host-derived proteins and identified key urinary protein biomarkers associated with *Schistosoma haematobium* infection, and offers new insights into host-parasite interactions and potential tools for non-invasive diagnostics. While validated in African pediatric populations from transmission hotspots, this host-protein approach inherently overcomes geographic limitations of parasite-based diagnostics; which is a critical advantage for surveillance in non-endemic regions where imported cases threaten gains toward elimination. These findings lay the groundwork for developing novel diagnostic approaches that could significantly improve the detection and surveillance of schistosomiasis, particularly in high-risk populations.

## Author summary

Schistosomiasis is a widespread disease caused by parasitic trematode flatworms, and affects millions of people, especially children in Africa. Detecting this disease is challenging because current methods require the microscopic identification of parasite eggs in urine; which is time-consuming and is not always accurate, especially in mild cases. Our study aims to improve diagnosis by discovering new host biomarkers that indicate parasite infection. We used proteomic method to screen urine samples from children in Zanzibar to identify infection-dependent host proteins. Machine learning methods emphasized five key host-derived proteins as candidate biomarkers. These proteins were validated using enzyme-linked immunosorbent assays (ELISA), confirming their potential for diagnosis of schistosomiasis. Our findings could lead to simpler and accurate tests that do not require microscopy. This would be a significant step forward in detecting and monitoring this disease, especially in high-risk communities. By improving diagnosis, we hope to reduce the burden of schistosomiasis and improve public health outcomes.

## Background

Schistosomiasis is a neglected disease, primarily within tropical and subtropical regions, and is caused by infection with Schistosoma parasites [1]. As of 2020, schistosomiasis was endemic in 78 countries, with 51 countries experiencing moderate to severe transmission [1]. Globally, an estimated 240 million people required preventive chemotherapy (PC) for schistosomiasis, including over 130 million school-aged children [2]. The African region bears the heaviest burden, and account for over 90% of cases requiring PC [2].

*Schistosoma haematobium* is unique among the schistosome species because it can manifest as urogenital schistosomiasis, particularly in Africa and the Middle East [2]. Human schistosomal infection occurs through contact with water contaminated by cercariae, the free-swimming larval stage released by infected snails, which can penetrate intact human skin [3]. Adult *Schistosoma haematobium* parasites inhabit the blood vessels of the bladder, and their eggs are excreted into urine; which in infected individuals often results in microscopic or visual hematuria, bladder fibrosis, dysuria, and kidney damage [1,4]. Chronic infections are associated with an elevated risk of bladder cancer [3,5,6]. Children aged 7–15 years exhibit the highest rates and intensities of infection [7], thus underscoring the urgent need for targeted interventions.

The current gold standard for diagnosing a *Schistosoma haematobium* infection is the detection of eggs through microscopic examination following filtration or centrifugation of urine [2,8]. However, the complex life cycle of schistosomes introduces significant diurnal and nocturnal variations in egg excretion, leading to considerable temporal inconsistencies in parasitological detection. Microscopic examination, though effective, requires specialized equipment and expertise, which limits its practicality in resource-constrained settings. Therefore, improved diagnostic methods are essential to achieve the goal of eliminating schistosomiasis as outlined in the Neglected Tropical Disease (NTD) Roadmap 2030 [1]. Although recent advances in DNA-based and antigen-antibody-based diagnostics for schistosomiasis offer improved sensitivity over traditional egg microscopy, significant challenges remain. DNA‐based methods such as qPCR, RPA, and LAMP require specialized laboratory equipment, costly reagents, lengthy procedures, and are prone to contamination during amplification. Antigen antibody assays, such as ELISA, POC‐CAA, and UCP‐LF CAA, are limited to detecting active infections and often lack the specificity needed to distinguish among Schistosoma species, while circulating antigen tests suffer from variable sensitivity across different formats, batch‐to‐batch inconsistencies, subjective readouts, and reduced accuracy in non‐endemic settings [9–12]. An urgent need exists for sensitive and reliable diagnostic tools tailored to different prevalence sites and schistosome species [1,13]. Host-derived biomarkers circumvent the logistical and biological limitations of parasite antigens. They do not require sourcing of live schistosomes or maintenance of recombinant protein platforms; thus, allowing assay development in any setting. Measured directly in urine or blood, these proteins reflect the integrated response of infected individuals to *Schistosoma haematobium* infection. Crucially, host candidate biomarkers identified in both experimental models and human cohorts have been consistently observed across diverse populations and stages of infection [14,15]. This reproducibility implies that the host response is largely independent of specific host or parasite genetic factors, thus supporting broad applicability. As pathology-related biomarkers, they uniquely mirror disease-induced tissue damage such as urothelial inflammation and fibrosis; thereby, facilitating both infection detection and clinical staging even in low-burden or post-clearance scenarios.

Mass spectrometry (MS)-based proteomics is widely used in clinical practice, and offers a powerful approach for disease diagnosis, biomarker discovery, and therapeutic guidance due to its high sensitivity, robustness, and high throughput [16–21]. The host immune response to parasite invasion induces distinct alterations in protein expression, which not only reflects host-parasite interactions but also provides diagnostic advantages by offering deeper insights into these interactions. Although parasite antigens for antibody-based assays can be produced through in vitro culture or recombinant DNA methods independent of endemic transmission, host-derived proteins offer a more practical diagnostic approach in non-endemic regions where parasite-derived material is scarce. The urinary proteins identified in *Schistosoma haematobium*-infected individuals are inherently specific to this parasite due to its exclusive residence in the urinary tract, thereby minimizing cross-reactivity with other schistosome species or unrelated pathogens. This specificity underpins their diagnostic utility and supports global applicability, even in settings with low or absent parasite burdens. Among biological fluids, urine is particularly advantageous for diagnostic studies as it is non-invasive, readily available in large quantities, and is reflective of systemic changes associated with disease [22]. Urinary proteomics has been successfully applied to identify new biomarkers for various diseases [23–25]. Additionally, machine learning has emerged as a key tool in medical studies [26], enabling precision medicine and advancing diagnostic accuracy [27]. The combination of proteomics and machine learning therefore provides a promising avenue to explore and differentiate diseases by identifying proteomic patterns associated with occurrence, progression, and prognosis [28,29].

Focusing on the high-risk population of school-aged children 7–15 years old, our study integrates data-independent acquisition (DIA) proteomics and machine learning to identify urinary host-derived proteins associated with *Schistosoma haematobium* infection. By screening for differential proteins, we seek to develop simple, non-invasive diagnostic methods tailored to this vulnerable group. Our findings have the potential to significantly enhance the prevention and control of schistosomiasis by enabling earlier and more accurate detection in high-risk populations.

## Materials and methods

### Ethics statement

The study was conducted under the management of the Pemba NTD office and was approved by the Zanzibar Ethics Review Board (ZAMREC002MAY014) and Jiangsu Institute of Parasitic Diseases (JIPD-2021–005). Verbal informed consent from the participants and written informed consent from their parents or guardians were obtained before the study.

### Study design, area, and population

**Fig 1:**
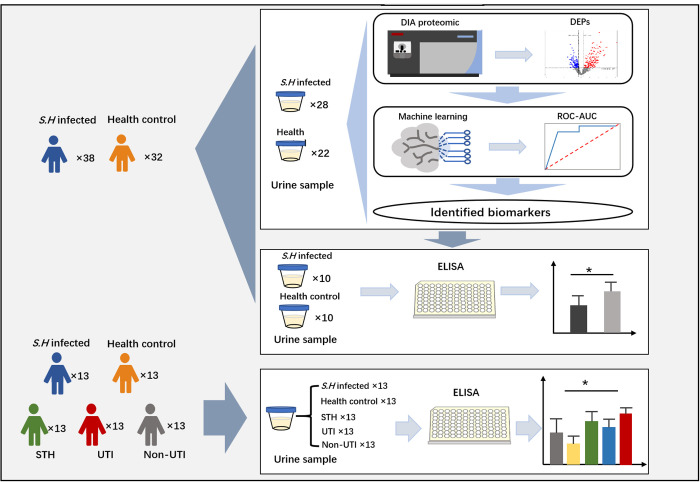
Key information about participants, research workflow, and data analysis. The illustration was drawn by hand using PowerPoint 2021 software.

This study was conducted within the framework of the China-Aid Project on Schistosomiasis Control Project in Zanzibar; which targets schistosomiasis-endemic regions of Zanzibar, Tanzania. Zanzibar is characterized by moderate to high endemicity of *Schistosoma haematobium* infection, particularly among school-aged children residing in rural communities such as Mtangani and Ukutini. Previous epidemiological data have documented ongoing transmission in these areas, and underscore the need for improved diagnostic methods. The study population comprised 135 children aged 7–15 years from Mtangani and Ukutini. Participants were recruited through local schools and community health centers with informed consent obtained from their guardians. The study design included a proteomic discovery cohort and two validation cohorts (A and B), all drawn from this target population. The proteomic cohort and validation cohort A included 70 children, of which 38 individuals were confirmed as positive for *Schistosoma haematobium* infection (infected group), and the remainder were 32 healthy individuals without infection or other comorbidities (control group). The validation cohort B comprised an additional 65 participants, which was divided equally into five clinically defined subgroups (n = 13 per group): infected, control, soil-transmitted helminth (STH), urinary tract infection (UTI), and non-UTI (Fig 1). Screening for *Schistosoma haematobium* infection was performed via microscopic examination of urine samples to identify parasite eggs. To ensure diagnostic accuracy each sample was independently examined in triplicate by three trained technicians. Participants with consistent detection of *Schistosoma haematobium* eggs across all examinations were assigned to the infected group. Individuals negative for *Schistosoma haematobium* eggs and without evidence of co-infections or other diseases were assigned to the control group.

The Kato-Katz technique was employed to detect helminth eggs in participant stool specimens freshly collected without contamination. For each sample, duplicate slides were prepared using a standardized 41.7 mg template, in which sieved stool was carefully loaded and leveled to ensure consistent thickness. A glycerol-malachite green-soaked cellophane strip was overlaid on the smear and gently compressed to achieve optimal transparency. The slides were left for 1 h to create glycerol-mediated transparency allowing visualization for the presence of hookworm eggs. After 24h the slides were examined by microscopy by two independent laboratory technicians for the presence of eggs of the soil-transmit helminth parasites *Trichuris trichiura, Ascaris lumbricoides,* and *Enterobius vermicularis*. Participants diagnosed with soil-transmitted helminth infections but without schistosomiasis were assigned to the STH group. Those with clinical and laboratory evidence of urinary tract infections but no parasitic infections were allocated to the UTI group. Finally, participants exhibiting symptoms unrelated to UTI and without parasitic infections were assigned as the non-UTI group. Individuals with co-infection with other parasites were excluded. Three independent technicians performed triplicate assessments on each subject. Urine samples (200 μL each) from the infected and control groups in the proteomic cohort and validation cohort A were lyophilized and stored at -80°C. Samples from all five groups in validation cohort B were collected and cryopreserved under the same conditions for further proteomic analyses.

### Sample preparation

The proteomic detection assays were carried out by the Mass spectrometry Research Platform of Shanghai University. Samples from the proteomic cohort and validation cohort A (70 samples in total) were redissolved in 200 μL of 8 M urea, followed by protein precipitation overnight using four volumes of precooled acetone at -20°C. After centrifugation at 12,000 × *g* for 20 minutes at 4°C, the precipitate was washed twice with 90% acetone. After removing the supernatant, the precipitate was air-dried, and resuspended in lysis buffer (1% SDS and 8 M urea). The supernatant after a second centrifugation was used to determine the protein concentration using a BCA Protein Assay Kit (Beyotime Biotechnology, Haimen, China), following the manufacturer’s instructions.

### Protein digestion

Protein denaturation, reduction, alkylation, tryptic digestion, and peptide clean-up were performed using an iST Sample Preparation kit (PreOmics, Planegg, Germany) according to manufacturer’s protocols. Briefly, 50 µL of lysis buffer containing the reducing agent dithiothreitol (DTT) and the alkylating agent iodoacetamide (IAA) was added to the sample, The samples were heated at 95°C for 10 minutes at 1,000 rpm with agitation to simultaneously denature proteins, reduce disulfide bonds, and alkylate free cysteine residues, thereby preventing reformation of disulfide bridges. After cooling to room temperature, trypsin digestion buffer was added, and the sample was incubated at 37°C for 2 hours at 500 rpm. Digestion was terminated with a stop buffer. Sample clean-up and desalting was conducted in an iST cartridge with recommended wash buffers, with peptides eluted in 2 × 100 µL of elution buffer, and then lyophilized using a SpeedVac (Thermo Fisher Scientific, Waltham, MA, USA).

### Nano-UHPLC-MS/MS analysis

The peptides were redissolved in solvent A (0.1% formic acid in water) and analyzed by an Orbitrap Fusion Tribrid mass spectrometer coupled to an EASY-nanoLC 1200 system (Thermo Fisher Scientific). A 3.5 μL peptide sample was loaded onto a 25 cm analytical column (75 μm inner diameter, 1.9 μm resin, Dr Maisch) and separated with a 60-minute gradient: 4% buffer B (80% acetonitrile with 0.1% trifluoroacetic acid) increasing to 30% after 53 min, then to 90% after 1 min, and maintained for 6 min. The column flow rate was 600 nL/min with a column temperature of 55°C. The electrospray voltage was set to 2 kV.

The mass spectrometer was operated in data independent acquisition (DIA) mode with a hybrid data strategy. A survey scan was conducted at 120,000 resolution with a normalized AGC target of 100% and a maximum injection time of 50 milliseconds (ms). DIA MS2 acquisition used 20 variable isolation windows following one full scan. MS2 resolution was set to 30,000, with a normalized AGC target of 400%, a maximum injection time of 72 ms, and normalized collision energy of 32.

### Data analysis

Raw DIA data were processed by Spectronaut 15.0 (Biognosys, Schlieren, Switzerland) with default settings. Database searches were conducted against the uniprot-homo sapiens (version201907, 20,428 entries), assuming trypsin as the digestion enzyme. Carbamidomethylation was set as a fixed modification, and oxidation as a variable modification. Retention time prediction was set to dynamic iRT, with extensive mass calibration and ideal extraction windows determined dynamically by Spectronaut. A Q value (FDR) cut-off of 1% was applied at both the precursor and protein levels. Decoy generation was set to mutated, involving random amino acid position swamps (min = 2, max = length/2). The normalization strategy was set to local normalization. The average top three filtered peptides that passed the 1% Q value cut-off were used to calculate the major group quantities. The proteins with≥50% of expression values in any group were retrained, while proteins with≤50% missing values were filled with the mean of samples in the same group. Data were transformed into log_2_ scale and median-normalized. Different expressed proteins (DEPs) were identified using a *t*-test (*p* value was < 0.05) and an absolute fold change >1.5. Visualization of the statistical analysis results were generated accordingly. To explore the biological functions associated with DEPs, GO pathway enrichment analysis was conducted using co-expression genes. The analysis criteria required the number of differentially expressed hetero-proteins in each pathway to range between 3 and 40. The results were prioritized based on statistical significance, with the top 10 pathways selected in ascending order of P-value.

### Machine learning

Machine learning was performed using Python (v3.9). Feature selection was conducted using the top 20 DEPs, Least Absolute Shrinkage and Selection Operator (LASSO) algorithm, and Support Vector Machine - Recursive Feature Elimination (SVM-RFE). The common proteins selected by all three methods were then used for subsequent classifier analyses. Based on these proteins, six machine learning models were trained using the scikit-learn package (v0.24.3): Bayesian model (Bys), logistic regression (LR), decision tree (DT), random forest (RF), support vector machine (SVM), and extreme gradient boosting (XGBoost). The optimal feature combination for each model was determined by the last-place exclusion method after ranking the feature importance. Samples were randomly divided into the training set and the test set at a ratio of 6:4, and cross-validation was performed using a stratified 5-fold split to achieve a robust estimate of model performance. To validate the classification performance of the ELISA data, these machine learning models were also applied. Initially, each feature was validated separately, followed by a combined validation of both features through concatenation. The data were standardized, and 10-fold Stratified K-Fold cross-validation was used to ensure consistent class distribution across folds. Model performance was evaluated by calculating ROC curves and AUC values to assess the ability to distinguish between different groups. Additionally, classification reports were generated to evaluate accuracy, precision, recall, and F1 score; which provided a comprehensive assessment of the ability of each model to correctly classify the samples.

### Protein abundance verification by ELISA

Protein expression in urine samples was verified using an enzyme-linked immunosorbent assay (ELISA). Urine samples and an ELISA kit (Elabscience Biotechnology, Wuhan Fine Biotech, and Shanghai Jianglai Industrial Limited by Share) were equilibrated to room temperature for 20 minutes prior to analysis. For standard wells, 50μL of standard solutions at varying concentrations were added, while for sample wells, 10μL of each test sample was combined with 40μL of sample dilution buffer. Blank wells were prepared without the addition of any sample. Subsequently, 100μL of horseradish peroxidase-conjugated detection antibody was introduced to all wells (blank, standard, and sample), and the plate was sealed with adhesive film before being incubated at 37°C for 60 minutes. After incubation, the liquid contents of the wells were discarded, and the plate was blotted dry using absorbent paper. Each well was then washed five times with washing solution, allowing it to stand for 1 minute during each wash. After washing, 50μL of substrate solutions A and B were added to each well and incubated in the dark at 37°C for 15 minutes. The reaction was terminated by adding 50 μL of stop solution to each well, and absorbance was measured within 15 minutes at 450 nm using a microplate reader. The proteins in each sample were determined using a standard curve derived from the OD values of the standard solutions.

### Statistical analysis

Statistical analysis was performed using SPSS 25.0 (IBM, Armonk, NY, USA) software. Age and egg counts, which did not follow a normal distribution, were presented as median [IQR]. Comparisons were performed using the Mann-Whitney U test for two groups or the Kruskal-Wallis H test for three or more groups. ELISA measurements were expressed as the mean ± standard deviation (SD). These data were analyzed using the independent samples t-test for two-group comparisons or one-way ANOVA for comparisons among multiple groups. For ANOVA, post hoc pairwise comparisons were adjusted using the Bonferroni method when overall significance was detected. All statistical tests were two-tailed, and a *p*-value<0.05 was considered statistically significant.

## Results

### Demographic characteristics

This study screened 135 urine samples, including 70 samples from *Schistosoma haematobium* infected individuals and healthy controls, divided into a proteomic cohort and validation cohort A, and an additional 65 samples categorized into five clinically defined groups in validation cohort B (infected, control, STH, UTI and non-UTI). The key methodological information is summarized in Fig 1 and the demographic parasitological data is summarized in Table 1. The Proteomic cohort included 24 males and 4 females in the infected group, with an average age of 9.00 [8.25, 12.00] years, while the control group included 14 males and 8 females with an average age of 9.00 [8.00, 10.00] years. The distribution according to gender did not differ significantly (*P* = 0.07), nor did age (*P* = 0.75). The Validation cohort A included 8 males and 2 females in both groups, with the average ages of 9.50 [8.50, 12.00] for the infected group and 9.00 [8.00, 10.00] years for the control group. No significant differences were found for gender (*P* = 1.00) or age (*P* = 0.56). In the Validation cohort B, the non-UTI group included 5 males and 8 females, whereas the remaining four groups each consisted of 7 males and 6 females. The median ages for these groups were as follows: infected group 11.00 [10.00, 12.00] years, control group 12.00 [9.00, 13.00] years, UTI group 11.00 [10.00, 12.00] years, STH group 8.00 [6.00, 12.50] years, and the non-TI group 10.00 [9.00, 12.00] years. No significant differences were observed in either age or gender distribution among the five groups (*P* = 0.094 and *P* = 0.91, respectively). Parasitological results indicated the presence of *Schistosoma haematobium* eggs in the urine within the infected group, none of whom exhibited visible hematuria. Egg counts ranged from 1 to 196 in the Proteomic cohort, with a median of 12.50 [7.25, 34.50], from 2 to 146 eggs in the Validation cohort A, with a median of 8.50 [3.00, 43.00], and from 1 to 160 eggs in the Validation cohort B with a median of 12.00 [7.50, 27.00]. Statistical analysis revealed no significant differences in egg counts among the infected groups across these cohorts (P = 0.61). As expected, no *Schistosoma haematobium* eggs were detected in the urine of the control group, and no hematuria was observed.

**Table 1:** Basic characteristics of the school aged participants in this study.

Cohort	Group	F:M ratio	Age (median [IQR])	No. of eggs	Independent reviews	Initial diagnosis
Proteomic cohort	Infected (n = 28)	4F:24M	9.00 [8.25,12.00]	12.50 [7.25,34.50]	3	Yes
	Control (n = 22)	8F:14M	9.00 [8.00,10.00]	–	3	–
Validation cohort A	Infected (n = 10)	2F:8M	9.50 [8.50,12.00]	8.50 [3.00,43.00]	3	Yes
	Control (n = 10)	2F:8M	9.00 [8.00,10.00]	–	3	–
Validation cohort B	Infected (n = 13)	6F:7M	11.00 [10.00,12.00]	12.00 [7.50,27.00]	3	Yes
	Control (n = 13)	6F:7M	12.00 [9.00,13.00]	–	3	–
	UTI (n = 13)	6F:7M	11.00 [10.00,12.00]	–	3	–
	STH (n = 13)	6F:7M	8.00 [6.00,12.50]	–	3	–
	Non-UTI (n = 13)	8F:5M	10.00 [9.00,12.00]	–	3	–

F = female; M = male.

UTI = Urinary tract infection group; STH = Soil-Transmitted Helminths infection group;

Non-UTI = non-urinary tract infection group

F:M Ratio: The ratio of females to males in each group;

Age (median [IQR]): Median age of participants with interquartile range (IQR);

No. of eggs (median [IQR]): Number of Schistosoma haematobium eggs detected in urine samples, expressed as median [IQR];

Independent reviews: Refers to the number of independent diagnostic assessments performed by trained technicians to ensure accuracy;

Initial diagnosis: Indicates whether participants were confirmed positive for Schistosoma haematobium infection during the initial screening phase (Yes/No).

### Proteomic screening of differential proteins

**Fig 2:**
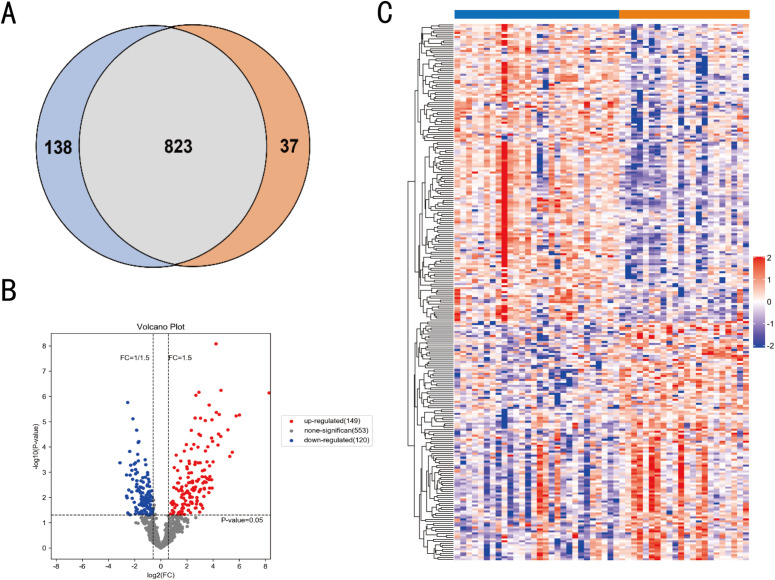
Proteomic profiling of urine from *Schistosoma haematobium* infected volunteers and controls. **(A)** Venn diagram illustrating the overlap of identified host proteins between the infection and control groups. **(B)** Volcano plot depicting changes in protein abundance between the two groups. A total of 269 DEPs (fold change ≥ ±1.5, p < 0.05) were identified. **(C)** Heatmap showing the expression levels of common proteins across all samples. Red and purple indicate higher and lower protein abundances, respectively, while blue and orange represent the infection (P) and control (N) groups, respectively.

Urinary proteomic data from the Proteomic cohort was used to identify signatures of *Schistosoma haematobium* infection. Although parasite proteins were detected in the urine tested, they were not further studied due to the lack of validation for protein identification. Host proteins from the control and infected groups were analyzed for differential expression. In total, using the DIA proteomic data 961 and 860 host proteins were identified in the infection and control groups (S1 Table), respectively. A Venn diagram showed that 823 host proteins were shared in both groups (Fig 2A), accounting for 85.64% and 95.70% of the trusted proteins in the infected and control groups, respectively. A constructed volcano plot revealed distinct differences in the protein profiles between the two groups (Fig 2B). Differential protein analysis determined that 269 proteins out of 823 common proteins were differentially expressed, of which 149 (FC > 1.5, *P* < 0.05) were up-regulated and 120 (FC < 0.67, *P* < 0.05) were down-regulated in the infected group (Fig 2B). We further conducted hierarchical clustering analysis using heatmaps to classify the differentially expressed host proteins between the two groups and to evaluate their expression patterns both within and across groups. As shown in Fig 2C, the variation trends of these DEPs distinctly separated the two groups, indicating their potential to effectively capture the impact of *Schistosoma haematobium* infection and their strong classification capability. Based on FC values, the top 20 proteins with the most significant up-regulation or down-regulation are listed in Table 2.

**Table 2:** List of the top 20 up- and down-regulated proteins.

Protein ID	Protein name	FC	*P* value	Change
P01023	Alpha-2-macroglobulin	313.315	7.26E-07	up
P68871	Hemoglobin subunit beta	64.859	5.42E-06	up
P69905	Hemoglobin subunit alpha	53.908	5.92E-06	up
P06310	Immunoglobulin kappa variable 2–30	44.363	0.000162	up
P02787	Serotransferrin	38.834	0.000236	up
P02647	Apolipoprotein A-I	34.938	2.1E-05	up
P37802	Transgelin-2	24.316	5.75E-07	up
P0C0L4	Complement C4-A	23.991	3.79E-05	up
P02652	Apolipoprotein A-II	22.034	5.15E-06	up
P0C0L5	Complement C4-B	21.250	3.06E-05	up
P27797	Calreticulin	20.682	8.35E-05	up
A0A0B4J1V6	Immunoglobulin heavy variable 3–73	19.038	4.26E-06	up
P01717	Immunoglobulin lambda variable 3–25	18.674	8.19E-09	up
P04180	Phosphatidylcholine-sterol acyltransferase	16.057	0.000385	up
P36955	Pigment epithelium-derived factor	15.574	5.98E-05	up
Q8N1N4	Keratin, type II cytoskeletal 78	15.184	0.004264	up
P00738	Haptoglobin	14.968	8.37E-06	up
P48740	Mannan-binding lectin serine protease 1	14.795	4.38E-05	up
P00450	Ceruloplasmin	14.693	0.001977	up
P01871	Immunoglobulin heavy constant mu	13.437	0.001486	up
P52272	Heterogeneous nuclear ribonucleoprotein M	0.115	0.000425	down
P07911	Uromodulin	0.163	0.001288	down
Q03167	Transforming growth factor beta receptor type 3	0.163	0.008765	down
Q5ZPR3	CD276 antigen	0.167	0.010316	down
Q9UMS6	Synaptopodin-2	0.172	1.74E-06	down
Q496F6	CMRF35-like molecule 2	0.174	0.040438	down
P07711	Cathepsin L1	0.183	0.005019	down
Q6UXB3	Ly6/PLAUR domain-containing protein 2	0.192	0.00015	down
P15814	Immunoglobulin lambda-like polypeptide 1	0.194	0.048292	down
P13645	Keratin, type I cytoskeletal 10	0.197	0.001304	down
P13646	Keratin, type I cytoskeletal 13	0.203	0.000524	down
P35908	Keratin, type II cytoskeletal 2 epidermal	0.213	0.006031	down
Q8N386	Leucine-rich repeat-containing protein 25	0.215	0.000842	down
P04222	HLA class I histocompatibility antigen	0.219	0.010701	down
P02538	Keratin, type II cytoskeletal 6A	0.222	0.00191	down
Q9BZJ0	Crooked neck-like protein 1	0.228	7.64E-06	down
P04746	Pancreatic alpha-amylase	0.244	0.000472	down
P19971	Thymidine phosphorylase	0.244	0.015565	down
P63261	Actin, cytoplasmic 2	0.258	0.045079	down
P19440	Glutathione hydrolase 1 proenzyme	0.261	0.000422	down

### DEP functional analysis

**Fig 3:**
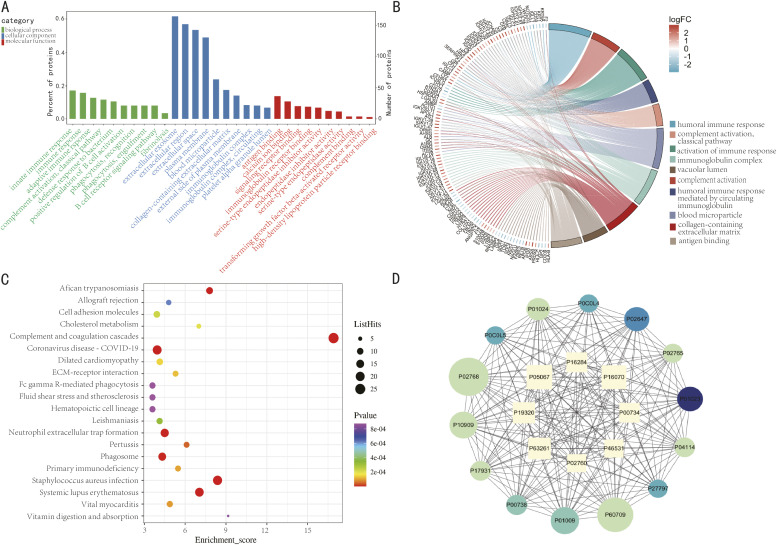
Bioinformatics analysis of DEPs. **(A)** GO analysis displaying the top 10 enriched GO terms across three major categories: biological process, molecular function, and cellular component. **(B)** GO enrichment analysis and chord diagram. The left side of the diagram lists the gene names of the proteins, with red indicating up-regulation and blue indicating down-regulation. The right side shows the GO terms enriched for these genes. **(C)** KEGG pathway analysis, highlighting the top 20 significantly enriched pathways. The x-axis represents the rich scores. The y-axis lists the KEGG pathway terms. The size of the dot indicates the number of proteins enriched in the pathway, and the color of the dots represents p-values. **(D)** Protein-protein interaction network analysis, showing the top 20 proteins ranked by connectivity. Characters represent protein IDs, circles indicate up-regulated proteins, and squares indicate down-regulated proteins.

GO enrichment analysis was performed on each cluster to determine the biological significance of these DEPs. They were categorized into three biological modules including gene ontology (GO), biological processes (BP), cellular composition (CC), and molecular function (MF) (Fig 3A). The top 10 most enriched GO terms of the differential proteins were highlighted, with key BP terms including innate immune response, complement activation, and defense response to bacterium; CC including extracellular exosome, extracellular region, and plasma membrane; and MF including antigen binding, calcium ion binding, and signaling receptor binding (Fig 3A). The co-expression genes linked to expression disparities exhibited significant enrichment in key BP, including humoral immune response, complement activation, activation of immune response, and humoral immune response mediated by circulating immunoglobulin (Fig 3B). To understand the biological pathways affected during infection, we used a bubble chart to visualize the enrichment profiles of DEPs across KEGG metabolic pathways, highlighting the significance of protein enrichment in various pathways. Among the top 20 highly enriched pathways were complement and coagulation cascades, *Staphylococcus aureus* infection, and neutrophil extracellular trap formation (Fig 3C). Additionally, Fig 3D depicts the 20 proteins with the most intricate interactions, including 14 up-regulated and 8 down-regulated proteins.

### Machine learning-based selection of biomarkers for classification of *schistosomiasis haematobium*

**Fig 4:**
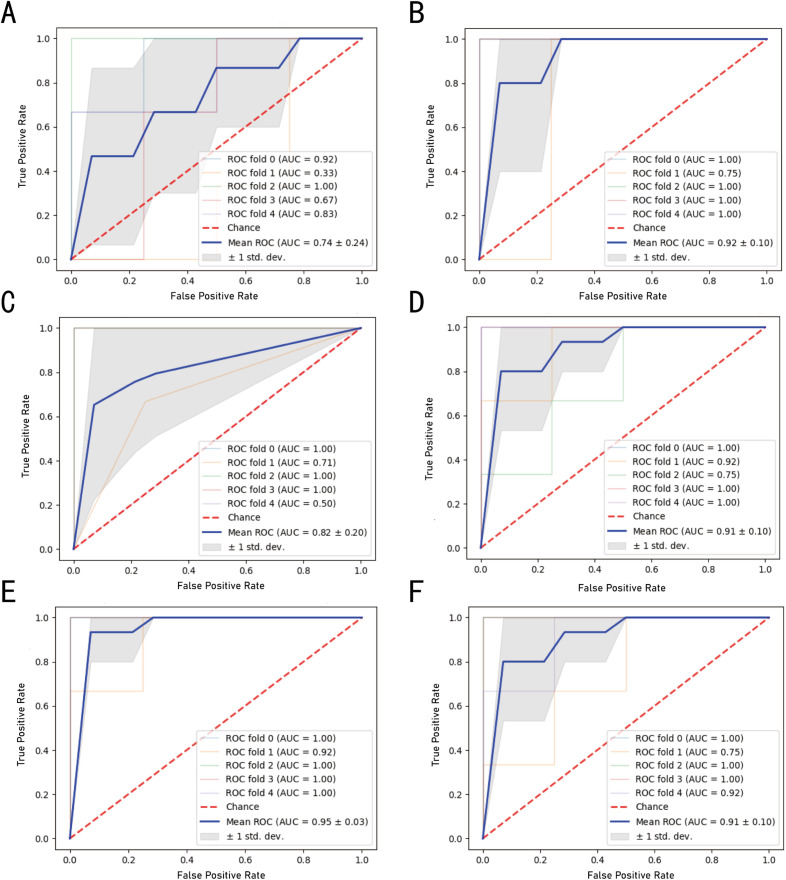
The area under the ROC curve (AUC) of cross-validation in each machine learning model. **(A)** Bayesian model (bys), **(B)** logistic regression (LR), **(C)** decision tree (DT), **(D)** random forest (RF), **(E)** support vector machine (SVM), and **(F)** extreme gradient boosting (XGBoost).

Machine learning was employed to identify biomarkers capable of distinguishing infected versus non-infected individuals. Using the urinary proteome data from the Proteomic cohort, six machine learning models were trained. The performance of each model was evaluated on the Validation cohort A, with accuracies of 57.14% for Bys, 88.57% for LR, 85.71% for DT, 71.43% for RF, 94.26% for SVM, and 91.42% for XGBoost. The area under the ROC curve (AUC) for each model is shown in Fig 4, with all models demonstrating AUC values above 90%.

Synaptopodin-2 (SYNPO2) and phosphatidylcholine-sterol acyltransferase (LCAT) were identified as important classification features in all models except the Bys model. CD276 antigen appeared in four models (LR, DT, RF, SVM), while heterogeneous nuclear ribonucleoprotein M (hnRNPM) and alpha-2-macroglobulin (α2M) were included in three models (DT, RF, XGBoost).

### Protein validation by ELISA

**Fig 5:**
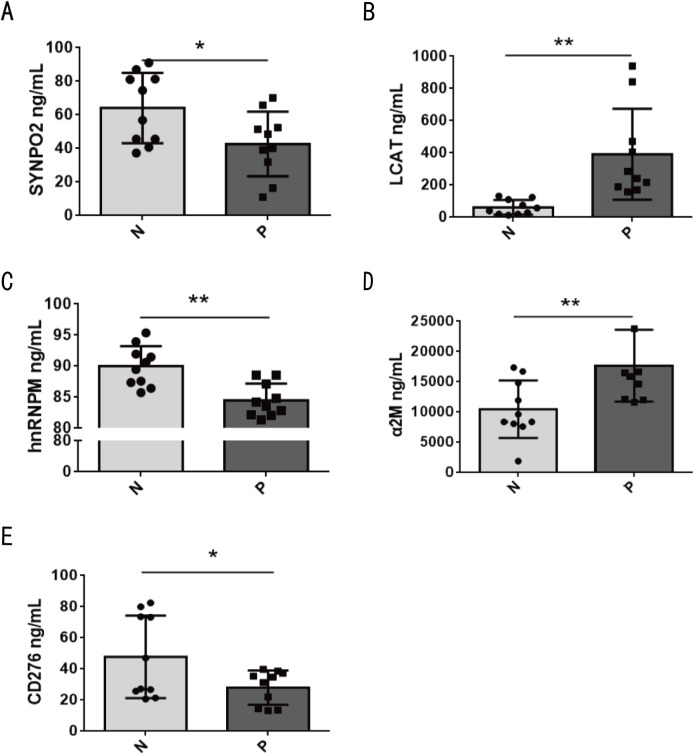
Validation of the selected DEPs between the control (N) and infection (P) groups by ELISA. (**A)** SYNPO2, **(B)** LCAT, **(C)** hnRNPM, **(D)** α2M, **(E)** CD276 antigen. Data are presented as mean ± SD. *P < 0.05, **P < 0.01.

**Fig 6:**
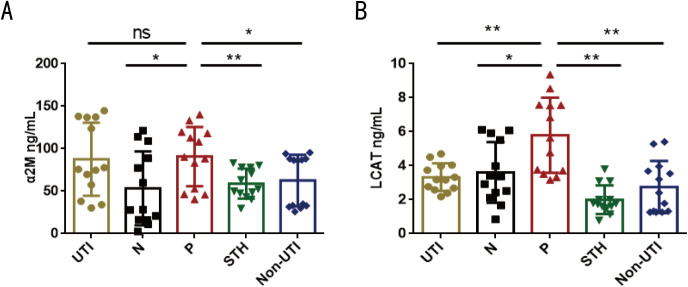
Validation of the selected DEPs among the five groups by ELISA. **(A)** α2M, **(B)** LCAT. Data are presented as mean **± **SD. Groups are defined as: P, *Schistosoma haematobium* infected group; N, control group; STH, soil-transmitted helminth infection group; UTI, urinary tract infection group; non-UTI, symptomatic individuals without UTI. The infected group (P) was compared with the other four groups. ^*^P < 0.05, ^**^P < 0.01.

To validate the findings from machine learning, ELISA was performed to detect the expression levels of candidate proteins in the validation cohort A. The expression levels of SYNPO2, CD276, hnRNPM, α2M, and LCAT exhibited significant differences between the infected and control groups (Fig 5). Specifically, the protein levels of SYNPO2 (FC = 0.66, *p* < 0.05), CD276 antigen (FC = 0.59, *p* < 0.05), and hnRNPM (FC = 0.94, *p* < 0.01) were markedly reduced in the urine of the infected children, while α2M (FC = 1.69) and LCAT (FC = 6.32) were significantly increased (*p* < 0.01) (Fig 5A–5E). Further experimental validation was performed in the Validation cohort B for α2M and LCAT, which were significantly increased in the infection group within the Validation cohort A. As revealed, the expression of α2M in the infection group was significantly higher than that in the control (FC = 1.70, *p* < 0.05), STH (FC = 1.54, *p* < 0.01) and non-UTI groups (FC = 1.45, *p* < 0.05), with no significant difference compared to the UTI group (FC = 1.04) (Fig 6A). Similarly, LCAT was significantly elevated in the infected group compared to the control (FC = 1.61, *p* < 0.05), STH (FC = 2.90, *p* < 0.01), UTI (FC = 1.74, *p* < 0.01) and non-UTI (FC = 2.11, *p* < 0.01) (Fig 6B).

### Machine learning-based model validation of ELISA data

The performance of individual biomarkers (LCAT and α2M) and their combinations was evaluated using six machine learning models: Logistic Regression, Random Forest, Support Vector Machine (SVM), Decision Tree, XGBoost, and Bayes, based on area under the curve (AUC), accuracy, precision, recall, and F1-score (see S2 Table for detailed metrics). Random Forest and XGBoost achieved near-perfect classification across all groups (control group, infection group, UTI and non-UTI), with 100% accuracy. However, due to the small sample size (65 samples), k-fold cross-validation was used to mitigate potential overfitting and ensure model generalizability.

While the Random Forest and XGBoost models demonstrated high accuracy, there was variability in precision and recall across different groups. For example, the SVM model showed lower performance in distinguishing between the UTI and Non-UTI groups, with precision ranging from 0.44 to 0.67 and recall from 0.31 to 0.62, suggesting that some categories were more difficult to classify due to less distinguishing biomarker expression. Combining the LCAT and α2M biomarkers led to a significant improvement in performance across all models. The combined model demonstrated better balance in classification metrics - precision, recall, and F1-score - compared to individual biomarkers; thus providing more reliable classification, particularly for groups where individual biomarkers showed greater variability.

**Fig 7:**
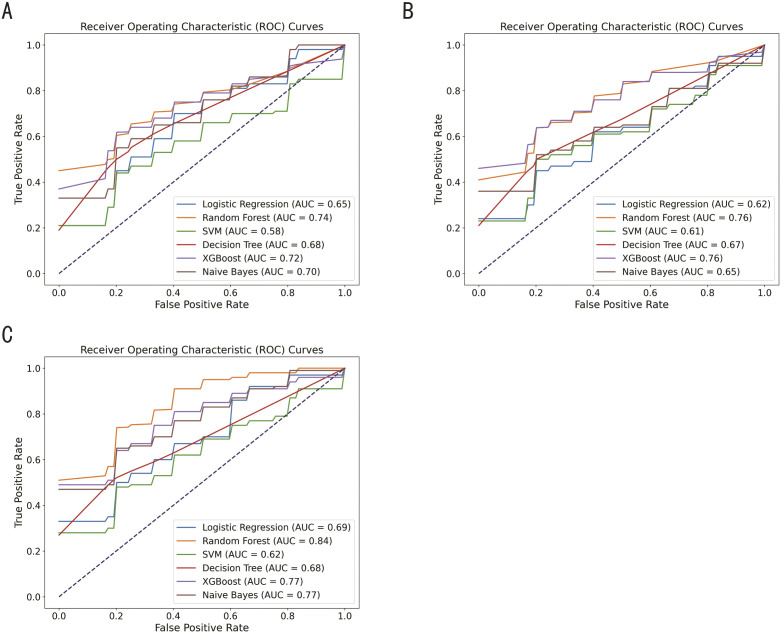
ROC curves evaluating the diagnostic performance of individual and combined biomarkers. Curves showing the diagnostic performance of each biomarker and their combination in distinguishing between the five groups: control **(N)**, infection **(P)**, soil-transmitted helminth (STH), urinary tract infection (UTI) and symptomatic individuals without UTI (non-UTI). **(A)** LCAT, **(B)** α2M, **(C)** the combined LCAT and α2M.

The ROC curves (Fig 7) showed that the combined biomarker model consistently outperformed individual biomarkers in terms of AUC, highlighting a stronger overall diagnostic ability.

## Discussion

*Schistosoma haematobium* infection remains a significant public health issue, particularly in sub-Saharan Africa. Traditional diagnostic methods rely on microscopy to detect parasite eggs in urine. However, this approach has inherent limitations, particularly when dealing with mild infections, where egg output fluctuates significantly and it is difficult to detect early or low-intensity infections. This necessitates repeated urine examinations, which can be labor-intensive and require significant expertise. Recent advances in MS-based proteomics and machine learning (ML) have provided new opportunities to identify disease-specific biomarkers in urine, offering the potential for a more sensitive, non-invasive diagnostic approach. This study represents the inaugural application of DIA proteomics in conjunction with machine learning in identifying novel urinary host-derived protein biomarkers for *Schistosoma haematobium* infections; and provides critical insights into the host-parasite interaction and offers a promising avenue for improving diagnostic sensitivity and accuracy. These results were consistent with our proteomics findings, validating the combined DIA-ML approach and underscoring the potential roles of these biomarkers in the pathogenesis of *schistosomiasis haematobium*.

Our study noted a significant up-regulation of α2M in the urine of individuals infected with *Schistosoma haematobium*. α2M is a major proteinase inhibitor that plays a critical role in controlling hemostasis by inhibiting thrombin and plasmin, thereby promoting clot rupture and increasing blood flow. These actions create an environment conducive to parasite survival within blood vessels [30–32]. This function is particularly important for schistosomes, which exert significant influence on the host hemostatic system [33]. Studies on other parasitic infections, such as *Trypanosoma*, also highlighted the protective role of α2M, enhancing macrophage phagocytosis and antimicrobial activity [32]. α2M may additionally help *Schistosoma haematobium* evade the immune system by modulating the complement system, which is typically involved in parasite eradication [30,32]. Increased α2M levels in infected individuals might reflect the ability of the schistosome to manipulate the host immune response, thereby facilitating its survival. While the elevated α2M levels in *Schistosoma haematobium* infected individuals are likely driven by parasite-mediated immune modulation to promote its survival, it is equally plausible that these increases reflect a host response to vascular damage and inflammation. α2M is an acute‑phase protease inhibitor that binds and modulates key angiogenic factors such as FGF‑2 and VEGF [34,35], enhances endothelial repair via FGF‑2/NO signaling [36], and promotes the shedding of pro‑angiogenic microvesicles from wound fibroblasts [37]. Such processes are not unique to schistosomiasis but are also observed in bacterial infections such as UTIs, where systemic inflammation and endothelial repair mechanisms could lead to α2M upregulation. This highlights the need for future studies to illuminate the contributions of schistosome-specific immune modulation versus general inflammatory responses in the context of α2M dynamics

ApoA1 was also identified in this study as a differential protein, and is a key protein component of high-density lipoprotein (HDL) [38]. HDL plays an essential role in host defense mechanisms, particularly in protecting against parasitic infections [39]. ApoA1 contributes to innate immune responses by enhancing pathogen clearance [39] and potentially serves as a biomarker for infections [40–44]. Previous studies have shown that ApoA1, as part of the trypanosome lytic factor (TLF), modulates the immune response to eliminate *Leishmania* and *Trypanosoma* [45–47]. A potential role of ApoA1 in *Schistosoma haematobium* infection could involve molecular mimicry, wherein the parasite exploits its immunomodulatory properties to evade detection by the host immune system [46]. Haptoglobin-related protein was identified as a differential protein in our study, and has been reported to be another active component in TLF [48]. TbHpHbR is a receptor for TLF1, which recognizes the complex formed by hemoglobin with haptoglobin (HP) or Hpr, and mediates heme uptake to facilitate parasite growth [47,49,50]. ApoA1 interacts with LCAT, influencing lipid metabolism and immune responses during infection; thereby suggesting a complex interplay between lipoproteins and immune modulation in schistosomiasis [51,52].

LCAT is an enzyme responsible for the synthesis of plasma cholesteryl esters and plays an essential role in the maturation of HDL [53]. LCAT activity is typically reduced during inflammatory processes [54], and studies have documented elevated urinary excretion of LCAT in nephrotic animals, accompanied by lower plasma levels [55]. This aligns with our findings in *Schistosoma haematobium* infection, where LCAT dysregulation may contribute to lipid metabolism abnormalities and inflammatory responses, ultimately supporting parasite survival. Studies on schistosomiasis mansoni have supported this result [56–58]. Additionally, LCAT deficiency is associated with abnormal lipid deposition in the kidneys, leading to renal dysfunction, which may further exacerbate the host immune and inflammatory response during infection [55,59].

In addition to α2M, ApoA1, and LCAT, several other proteins identified in this study, including the CD276 antigen and hnRNPM, which play key roles in immune modulation. CD276 antigen, known as the B7 congener 3 protein (B7-H3), is an immune checkpoint molecule that plays both co-stimulatory and co-inhibitory roles in the immune system [60,61]. CD276 has been involved in tumor immunity [60,62], and has been studied as a potential target for cancer immunotherapy [60,63–65]. In non-malignant tissues or infection, CD267 may act to inhibit T cell activation and proliferation [63], thus allowing the parasite to evade immune detection and survival in the host. HnRNPM is a pre-mRNA binding protein and part of the spliceosome complex [66], and has been proposed to be a host target in regulating viral infection [67–69]. HnRNPM influences cancer development through a variety of mechanisms, and potentially serves as a cancer marker and anti-cancer target [70–75]. Studies have shown that hnRNPM played a unique role as CEAR in various cells, such as Kupffer cells, other terminally differentiated cells, and certain cancer cells, with its binding to CEA triggering inflammation response [76]. HnRNPM inhibits part of the innate immune transcription in macrophages, and its deficiency induces expression of inflammatory and antimicrobial genes after innate immune stimulation [77]. Although CD276 and hnRNPM have roles in immune modulation and cancer, their observed patterns in individuals infected with *Schistosoma haematobium* in our study indicate a potentially specific involvement in this infection. Future studies are needed to validate these proteins across broader cohorts and investigate their expression in other endemic parasitic infections to confirm their specificity.

The actin binding protein SYNPO2 was also identified in our study [78]. SYNPO2 is known to be associated with nephrotic syndrome and is detectable in the cytoplasm of glomerular mesangial cells [79]. Mounting studies have indicated that low levels of SYNPO2 may be linked to the development and metastasis of cancer [80–83], suggesting that SYNPO2 might be a potential prognostic biomarker and new therapeutic target. In the context of *Schistosoma haematobium* infection, the down-regulation of SYNPO2 observed in our study may reflect a protective role against parasite evasion of the immune response. These findings suggest that SYNPO2 might serve as a potential prognostic biomarker for schistosomiasis.

In addition to critical biomarkers identified using machine learning, we also observed other potential proteins of interest, such as Tamm-Horsfall protein (THP), also called uromodulin. THP is the most abundant protein in normal urine, and is essential in urinary and systemic homeostasis [84]. Available data suggests that this protein may play a role in regulating urinary tract infections and immunomodulation [85]. Decreased urinary THP production is an effective indicator of tubular damage and decreased clearance of proinflammatory cytokines [86]. THP was identified as a marker of *Schistosoma mansoni* infection in children [87], and the results in our study showed that *schistosomiasis haematobium* also affected the THP levels in urine.

Our study underscores the importance of employing a multi-biomarker approach in diagnosing *Schistosoma haematobium* infection. The combination of biomarkers provides a more reliable and effective diagnostic tool compared to solely using single markers. The enhanced diagnostic performance observed with the combined biomarkers suggests the potential of a multi-biomarker strategy to improve disease detection and overcome some of the limitations of traditional egg-based detection methods. These findings emphasize the value of a multi-biomarker approach in enhancing classification robustness and providing more accurate diagnostic outcomes; thus supporting further investigation of such models to improve diagnostic accuracy and clinical decision-making in *Schistosoma haematobium* infection and related urinary tract infections.

While our study provides valuable insights into the urinary proteomics of *Schistosoma haematobium* infection, several limitations must be acknowledged. While host‑derived proteins offer practical advantages, such as ease of detection and reflection of tissue pathology, they lack parasite specificity and may overlap with markers of other inflammatory or neoplastic conditions. Future validation against diverse disease cohorts, including cancer and autoimmune disorders, will be critical to ensure schistosome specificity and diagnostic reliability. Although no visible hematuria was observed in any of the study participants, the potential presence of occult hematuria cannot be excluded. Occult hematuria may influence the expression of certain proteins, thereby affecting their specificity of *Schistosoma haematobium* infection. Future studies should incorporate diagnostic tests for occult hematuria to clarify its role in differential protein expression. In addition, the proteomic and bioinformatics techniques used in this study, while advanced, still have inherent limitation. For example, this study did not include comparative analyses with existing biomarkers used in traditional diagnostic methods, such as dipstick assays. Future studies should prioritize such comparisons to validate the diagnostic utility of newly identified proteins and explore the integration of these biomarkers into a multi-marker diagnostic panel. This approach could significantly enhance diagnostic sensitivity and specificity, especially in field-adapted setting.

These limitations are being actively addressed through our ongoing longitudinal project in Zanzibar, which has now entered its second phase with expanded partnerships between local health authorities, the WHO, and the developers of portable diagnostic devices. Future research will validate biomarkers in diverse cohorts, including those with other inflammatory or neoplastic conditions, and integrate parasite-derived peptides to enhance specificity. Multi-marker approaches will be explored to refine diagnostic accuracy and to address overlaps in host protein expression. Our team is currently implementing a follow-up study in Pemba, directly integrated with the China-Aid Schistosomiasis Control Project. This scaled-up effort will not only validate biomarker stability across transmission seasons but also pilot field-adapted urine collection protocols compatible with rural health posts. Investigating the functional roles of these proteins in the host-parasite interaction is essential for understanding how they contribute to infection pathogenesis. Assessing the correlation between biomarker levels and infection severity such as egg counts or clinical symptoms, will provide further insights into their diagnostic and prognostic value. Future research could also explore the integration of both parasite-derived and host-derived proteins into a unified diagnostic panel, which would provide a more comprehensive and effective diagnostic tool for detecting *schistosomiasis haematobium* and other neglected tropical diseases.

## Conclusions

This study demonstrated the feasibility of using urinary proteomics in conjunction with machine learning to identify biomarkers for *Schistosoma haematobium* infection. Our results provided a comprehensive urinary protein profile for people at high risk of *Schistosoma haematobium* infection*,* and identified the proteins SYNPO2, LCAT, CD276 antigen, hnRNPM, and α2M as potential diagnostic markers that could improve the sensitivity and specificity of *Schistosoma haematobium* detection. LCAT and α2M were further validated using ELISA and machine learning, supporting the utility of combining multiple biomarkers for improved diagnostic performance. Host-derived biomarkers reflect the host’s integrated response to infection, offering unique insights into host-parasite interactions and associated tissue damage. Unlike parasite antigens, they eliminate the need for live schistosomes or recombinant protein platforms, enabling broader applicability and improved diagnostic performance across diverse settings. By expanding this approach to larger cohorts and refining our methodology, we hope to contribute to the development of more reliable and non-invasive diagnostic tools for schistosomiasis haematobium disease, ultimately advancing both the diagnosis and treatment for infection with this prevalent parasite.

## Supporting information

S1 Table Host proteins identified by DIA proteomic analysis. Contains two sub-tables: **(A) The positive group:** List of 961 host proteins and their expression levels detected in *Schistosoma haematobium* infected samples. **(B) The negative group:** List of 860 host proteins and their expression levels detected in healthy control samples.
(XLSX)

S2 Table Classification reports for machine learning models. Contains three sub-tables of model performance metrics (AUC, accuracy, precision, recall, F1‑score) for six algorithms (Logistic Regression, Random Forest, SVM, Decision Tree, XGBoost, Bayes): **(A) LCAT:** Performance when using only LCAT as the input biomarker. **(B) α2M:** Performance when using only α2M as the input biomarker. **(C) Combined:** Performance when using LCAT and α2M together.
(XLSX)

## Abbreviations

DIA: data independent acquisition
NTD: neglected tropical disease
PC: preventive chemotherapy
SH: Schistosoma haematobium
MS: mass spectrometry
Bys: bayesian model
LR: logistic regression
DT: decision tree
RF: random forest
SVM: support vector machine
XGBoost: extreme gradient boosting
ELISA: enzyme-linked immunosorbent assay
SYNPO2: synaptopodin-2
LCAT: phosphatidylcholine-sterol acyltransferase
hnRNPM: heterogeneous nuclear ribonucleoprotein M
α2M: alpha-2-macroglobulin
Apo AI: Apolipoprotein AI
TLF: Trypanosome lytic factor
HP: haptoglobin
THP: Tamm-Horsfall protein
